# Whole-Genome Sequences of Corynebacterium macginleyi CCUG 32361^T^ and Clinical Isolates NML 080212 and NML 120205

**DOI:** 10.1128/MRA.01506-18

**Published:** 2018-12-13

**Authors:** Anne-Marie Bernier, Kathryn Bernard

**Affiliations:** a Department of Biology, Université de Saint-Boniface, Winnipeg, Manitoba, Canada; b National Microbiology Laboratory (NML)–CSCHAH Site, Public Health Agency of Canada, Winnipeg, Manitoba, Canada; c Department of Medical Microbiology, University of Manitoba, Winnipeg, Manitoba, Canada; University of Maryland School of Medicine

## Abstract

Draft genome sequences of Corynebacterium macginleyi CCUG 32361^T^ and clinical isolates NML 080212 and NML 120205 were assembled and studied. Genome sizes ranged from 2.35 Mb to 2.42 Mb, with G+C contents ranging from 57.1% to 57.2%.

## ANNOUNCEMENT

The lipophilic species Corynebacterium macginleyi, an ocular pathogen for some coryneforms provisionally called CDC group G-1, was described by Riegel et al. in 1995 (1). The first bilateral eye infection caused by this agent in Canada (NML 080212) was reported in 2010 (2). Upon review, the NCBI genome database was found to lack whole-genome sequence (WGS) data for this species. Therefore, in this study, C. macginleyi CCUG 32361^T^, previously acquired from the Culture Collection of the University of Gothenburg (CCUG; NML 080212), and a second clinical isolate recovered from an ocular infection in Vancouver, British Columbia (NML 120205) were characterized by WGS.

Bacteria were subcultured after storage at −80°C in Microbank vials (PRO-LAB) from NML stocks or from lyophilized CCUG 32361 and passed twice on Colombia blood agar (CBA) plates for 24 h at 35°C in 5% CO_2_. A loopful of plate culture was grown aerobically in Trypticase soy broth (BD) for 18 h at 35°C, and DNA was extracted from broth cultures using the DNA minikit (Qiagen). Paired-end whole-genome shotgun libraries were constructed using the Nextera XT library preparation kit, and samples were run separately for sequencing on the MiSeq 600-cycle kit (version 3) on a MiSeq sequencer (Illumina, Munich, Germany). Read quality was assessed with FastQC version 0.11.8 (http://www.bioinformatics.babraham.ac.uk/projects/fastqc/), and short paired-end reads were then merged with Fast Length Adjustment of SHort reads (FLASH, version 1.2.9) (3). Reads were then assembled using SPAdes (version 3.9.0) (4) with default settings. Sequencing and assembly metrics for the three strains are presented in Table 1. The assemblies produced draft genomes composed of 2,349,818 to 2,419,073 bp, with an average G+C content of 57.1% and an average coverage of 100-fold. Contigs from assembled shotgun data were annotated for genes and other features using Prokka (version 1.13) (5), which revealed an average of 2,321 coding DNA sequences (CDS). One clustered regularly interspaced short palindromic repeat (CRISPR) sequence with 11 spacers was found only in the genome of strain NML 080212 and was otherwise absent from the other two strains. Prophages were not found in any of the three genomes using Phaster (6).

**TABLE 1:** *Corynebacterium macginleyi* sequencing, assembly, and annotation metrics

Strain	No. of reads	No. of bases	No. of contigs >1 kb	Genome size (bp)	Coverage (×)	Contig *N*_50_ (bp)	G+C content (%)	No. of CDS (Prokka)
CCUG 32361^T^	391,644	116,274,866	56	2,419,073	96	128,157	57.1	2,366
NML 080212	462,220	134,561,054	77	2,417,567	95	57,952	57.09	2,337
NML 120205	477,946	136,596,203	40	2,349,818	109	163,397	57.21	2,260

Average nucleotide identity values using BLAST+ (ANIb) as calculated using JSpeciesWS (7) were used to compare the NML strains to CCUG 32361^T^ and to each other. All ANIb scores were greater than 98.74%. Similarly, the *in silico* DNA-DNA hybridization (*is*DDH) values as calculated using formula 2 of the Genome-to-Genome Distance Calculator (8) were greater than 90.2%. Isolates lacked fatty acid synthase genes, which were previously observed for lipophilic *Corynebacterium* species (9), but genes associated with mycolate synthesis were detected (10).

### Data availability.

Draft genome sequences of Corynebacterium macginleyi CCUG 32361^T^, NML 080212, and NML 120205 were deposited at DDBJ/ENA/GenBank under accession numbers REGE00000000, REGD00000000, and REGC00000000, respectively. The versions described in this paper are REGE01000000, REGD01000000, and REGC01000000, respectively. Sequence Read Archive accession numbers are SRX4938091, SRX4938092, and SRX4938093.
